# MEASURING NATIONAL TRENDS IN SOCIAL ISOLATION AND LONELINESS AMONG OLDER ADULTS AND THEIR CAREGIVERS

**DOI:** 10.1093/geroni/igae098.0800

**Published:** 2024-12-31

**Authors:** Mary Louise Pomeroy, Yiqing Qian, Katherine Ornstein

**Affiliations:** Chair; Co-Chair; Discussant

## Abstract

Social isolation and loneliness have negative implications for the health and care of older adults. Few studies have examined longitudinal patterns of social isolation and loneliness among older adults, or how these experiences are shared by caregivers and personal care networks. This research symposium uses various survey data methods to highlight trends in social isolation, loneliness, and care isolation among older adults and their caregivers. First, we report on trends in social isolation and loneliness over time. Leveraging six waves of data from the Longitudinal Ageing Study Amsterdam (LASA), Dr. Mehrabi describes the temporal sequence of social isolation, loneliness, and frailty across 21 years among a national sample of older adults from the Netherlands. Dr. Umoh uses the National Health and Aging Trends Study (NHATS) to describe trends in intermittent and persistent social isolation among older Americans between 2011–2021. Next, we examine social isolation and loneliness in the context of caregiving. Dr. Clair reports on personal care networks and care isolation among family and friends caring for older adults with Type II diabetes. Dr. Qian uses the National Study on Caregiving (NSOC) to examine prevalence and correlates of social isolation and loneliness among family and unpaid caregivers of older adults. Dr. Pomeroy concludes the session with a discussion of the strengths and challenges of using national survey data to measure social isolation, offering methodological strategies for handling missing data and proxy reports for persons with dementia. Dr. Ornstein, as discussant, synthesizes the implications for policy, practice, and future research.

